# Association between metabolic healthy obesity and female infertility: the national health and nutrition examination survey, 2013–2020

**DOI:** 10.1186/s12889-023-16397-x

**Published:** 2023-08-10

**Authors:** Jing Tang, Yun Xu, Zhaorui Wang, Xiaohui Ji, Qi Qiu, Zhuoyao Mai, Jia Huang, Nengyong Ouyang, Hui Chen

**Affiliations:** 1 Reproductive Medicine Center, Sun Yat-Sen Memorial Hospital, Sun Yat-Sen University, Guangzhou, People’s Republic of China; 2 Endocrinology Department, Sun Yat-Sen Memorial Hospital, Sun Yat-Sen University, Guangzhou, People’s Republic of China; 3 Department of Endocrinology, The Sixth Affiliated Hospital of Sun Yat-sen University, Guangzhou, People’s Republic of China; 4 Sun Yat-Sen Memorial Hospital, No. 107 Yanjiang West Road, Guangzhou, 510120 People’s Republic of China

**Keywords:** Metabolic healthy obesity, Infertility, National Health and Nutrition Examination Survey

## Abstract

**Background:**

Obesity has been confirmed to be associated with infertility. However, the association between metabolically healthy obesity (MHO), a subset of obesity with no metabolic abnormalities, and female infertility has not yet been investigated. This study aimed to examine the association between MHO and the risk of female infertility among United States.

**Methods:**

This study utilized a cross-sectional design and included 3542 women aged 20–45 years who were selected from the National Health and Nutrition Examination Survey (NHANES) 2013–2020 database. The association between MHO and the risk of infertility was evaluated using risk factor–adjusted logistic regression models.

**Results:**

Higher BMI and WC were associated with increased infertility risk after adjusting for potential confounding factors (OR (95% CI): 1.04(1.02, 1.06), *P* = 0.001; OR (95% CI): 1.02 (1.01, 1.03), *P* < 0.001; respectively). After cross-classifying by metabolic health and obesity according to BMI and WC categories, individuals with MHO had a higher risk of infertility than those with MHN (OR (95% CI): 1.75(0.88, 3.50) for BMI criteria; OR (95% CI): 2.01(1.03, 3.95) for WC criteria). A positive linear relationship was observed between BMI/WC and infertility risk among metabolically healthy women (*P*_non−linearity_=0.306, 0.170; respectively).

**Conclusions:**

MHO was associated with an increased risk of infertility among reproductive-aged women in the US. Obesity itself, regardless of metabolic health status, was associated with a higher infertility risk. Our results support implementing lifestyle changes aimed at achieving and maintaining a healthy body weight in all individuals, even those who are metabolically healthy.

**Supplementary Information:**

The online version contains supplementary material available at 10.1186/s12889-023-16397-x.

## Introduction

Infertility is the failure to achieve a pregnancy after 12 months or more of regular unprotected sexual intercourse defined by the International Committee for Monitoring Assisted Reproductive Technology [1]. The estimated prevalence of infertility among women of reproductive age in the United States is 15.5% and continues to increase at an annual rate of 0.37% [2, 3]. Infertility not only imposes a considerable financial burden on patients [4] and the healthcare system but also leads to psychological distress, including depression and anxiety disorders [5, 6]. Moreover, in some developing countries, female infertility can lead to discrimination, domestic violence, and social stigma [7, 8]. Therefore, identifying risks factors for infertility and developing preventive strategies are necessary to mitigate the adverse effects and social burden of infertility.

Obesity (defined as body mass index (BMI) ≥ 30 kg/m^2^) has emerged as a severe global health issue, particularly in the United States, with an age-adjusted prevalence of 42.4% [9]. Among women of reproductive age, obesity is also increasingly prevalent [10]. Various factors contribute to female infertility, and obesity has received substantial research interest [11].

Obesity often coexists with metabolic abnormalities, and obesity-related metabolic disorders can mediate obesity-related morbidity [12]. However, a portion of obese individuals exhibit few or no metabolic anomalies, a condition known as metabolically healthy obesity (MHO) [13]. The MHO phenotype can have distinct morbidity outcomes than not just a metabolically unhealthy phenotype but also a metabolically healthy normal weight (MHN) phenotype [14]. Prior research over the past decade suggests that those with MHO may be at an elevated risk of cardiovascular disease and cancer than those with MHN [14, 15], implying that the MHO phenotypes may not be a relatively benign condition in terms of these diseases. However, the relationship between MHO and female infertility has not been fully investigated. A well-designed study could provide compelling evidence proof regarding the association between MHO and female infertility, facilitate identification of high-risk populations, and assist in preventing female infertility.

Obesity can be defined in multiple dimensions. The body mass index (BMI) remains the most commonly used, widely accepted, relatively simple, and inexpensive measure of overweight and obesity. However, individuals with the same BMI can have markedly different fat distributions [16]. Central obesity’s potential role in reproductive health has garnered increasing interest recently [17, 18]. Waist circumference (WC), a simple measurement of central obesity, is the most common indicator of abdominal fat [19].

Therefore, the primary aim of this study was to investigate the association between MHO and female infertility risk utilizing multi-ethnic data from a nationally representative sample of US women. Two measures were used to define obesity: BMI as a measure of overall obesity and WC as a measure of central obesity.

## Materials and methods

### Data source and sample design

The original data were obtained from the National Health and Nutrition Examination Survey (NHANES). NHANES collects information on the health and nutrition status of a representative sample of the noninstitutionalized civilian population every two years using a complex, multistage, and stratified sampling design. National Health and Nutrition Examination Survey was approved by the National Center for Health Statistics Institutional Review Board and all participants signed an informed consent.

### Population

**Fig. 1:**
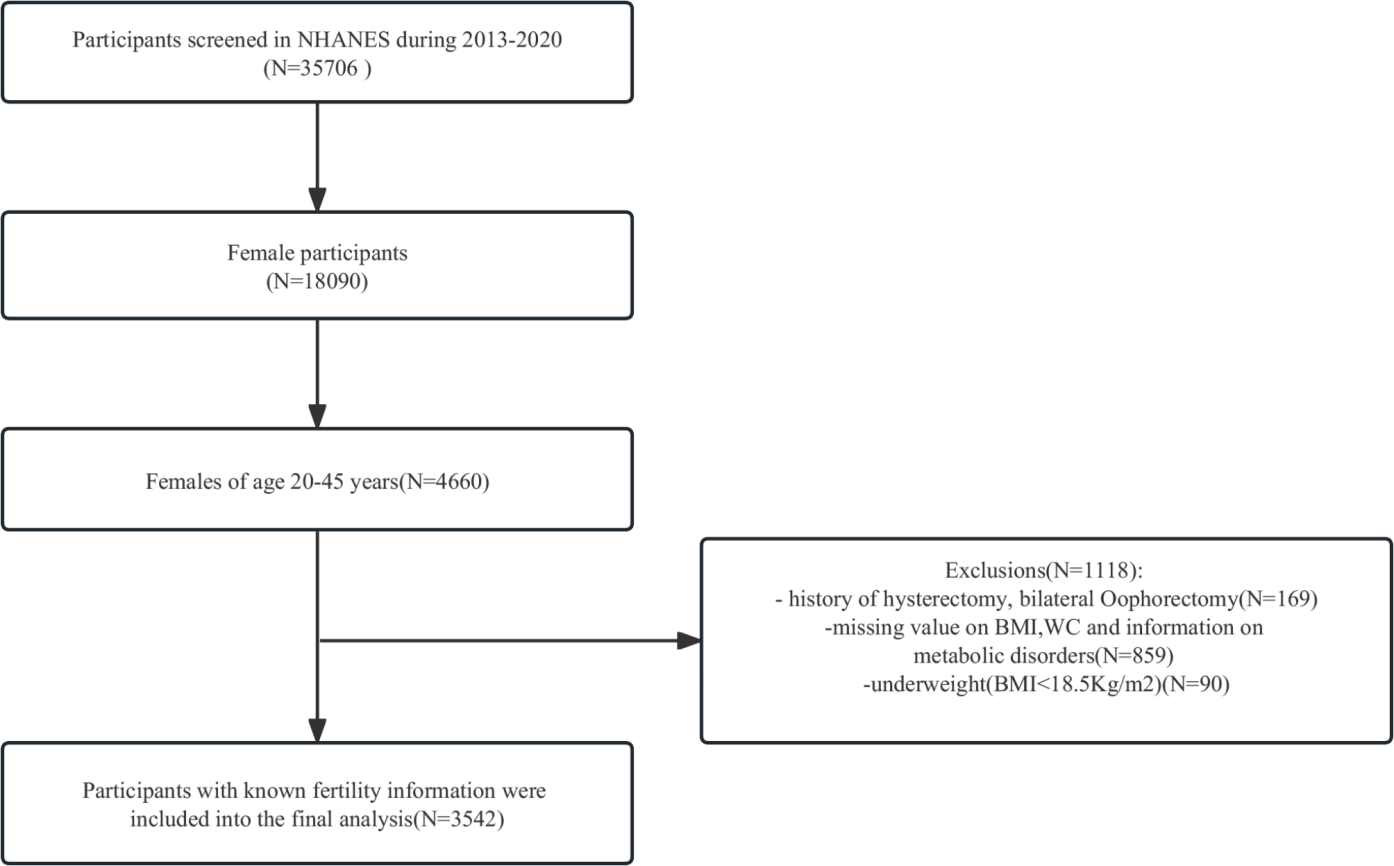
Flow chart of sample selection from the NHANES 2013–2020

This study utilized four consecutive cycles (2013–2020) of NHANES because only these cycles included a reproductive health questionnaire with questions on infertility. The inclusion criteria were (1) Female participants aged 20–45. (2) Female participants with complete fertility and infertility information. The exclusion criteria were (1) Female participants with a history of hysterectomy, or bilateral oophorectomy. (2) Female participants with missing values for BMI, WC, and information on metabolic disorders. (3) Female participants with BMI < 18.5 Kg/m^2^. After inclusion and exclusion criteria, we enrolled a total of 3542 participants (Fig. 1).

### Exposures and outcome

Our primary outcome was self-reported infertility from the Reproductive Health Questionnaire (question RHQ074): “Have you ever attempted to become pregnant over a period of at least a year without becoming pregnant?“ Those who answered “yes” were labelled “ever infertile,“ whereas women who answered “no” were labelled “fertile.“

Obesity was defined using BMI and WC. BMI was obtained by the body mass in kilograms divided by height in meters squared. Weight, height and WC were measured by the professional personnel. For BMI criteria, normal weight (BMI: 18.5–24.9 kg/m^2^), overweight (BMI: 25.0–29.9 kg/m^2^), and obese (BMI: ≥30 kg/m^2^) were categorized according to WHO guidelines [20]. For WC criteria, according to Lean ME et al. [21], central overweight was defined as WC ≥ 80 cm, and central obesity was defined as WC ≥ 88 cm for females.

Based on the 2009 harmonized criteria of metabolic syndrome [22],participants without any of the following four metabolic syndrome components were considered metabolically healthy: (1) Systolic blood pressure ≥ 130 mmHg or diastolic blood pressure ≥ 85 mmHg or self-reported hypertension or use of antihypertensive medication (2) Fasting blood glucose ≥ 5.6 mmol/L or self-reported diabetes or use of antidiabetic medication (3) HDL cholesterol < 1.29 mmol/L for women or use of lipid-lowering medication (4) Triglycerides ≥ 1.7 mmol/L or use of lipid-lowering medication. Those with one or more of the above components were classified as metabolically unhealthy [23].

Based on BMI criteria, participants were categorized into six phenotypes: metabolically healthy normal weight (MHN), metabolically healthy overweight (MHOW), metabolically healthy obese (MHO), metabolically unhealthy normal weight (MUN), metabolically unhealthy overweight (MUOW), and metabolically unhealthy obese (MUO). Similarly, according to WC criteria, participants were also categorized into the same six phenotypes: MHN, MHOW, MHO, MUN, MUOW, and MUO [23].

### Covariates

Potential confounders and effect modifiers were identified from previous literature and incorporated into a directed acyclic graph,which guided our modeling strategy. (Table 1, Supplemental Fig. 1). The participants’ age was the age at which they completed the survey. Race and ethnicity were classified into “White,“ “Black,“ and “other races.“ Marital status was classified as “Married or Living with a partner” or “Living alone.“ Education level was divided into three categories: “Less than high school,“ “High school,“ and “more than high school.“ Our study also considered the ratio of family income to poverty (PIR), drinking status (at least 12 drinks of alcoholic beverages in the last year), smoking status (according to the criteria of at least 100 cigarettes/year divided into current smoking, former smoking and never smoking), and prior pregnancy. In addition, some research indicates that physical activity may boost the likelihood of conception in infertile women. Hence, we categorized leisure time physical activity into three groups: “the inactive group (no leisure-time physical activity)”; “the moderately active group ( leisure time moderate activity 1–5 times per week with MET ranging from 3 to 6 or leisure-time vigorous activity 1–3 times per week with MET > 6)”;“the vigorously active group (those who had more leisure-time moderate-or-vigorous activity than the above)“ [24].

**Table 1:** Baseline characteristics of participants

Characteristics	Total (n = 3542)	Fertility (n = 3113)	Infertility (n = 429)	*P* value
Demography				
BMI	29.51(0.20)	29.14(0.22)	32.12(0.67)	< 0.001
WC	96.02(0.48)	95.01(0.51)	103.17(1.41)	< 0.001
Age, years	32.09(0.20)	31.66(0.20)	35.13(0.44)	< 0.001
PIR	2.70(0.06)	2.66(0.06)	2.93(0.11)	0.016
MHO-BMI				< 0.001
MHN	220(6.21)	195(6.48)	25(4.93)	
MHOW	252(7.11)	233(6.72)	19(3.73)	
MHO	433(12.22)	363(10.87)	70(15.39)	
MUN	928(26.2)	838(30.25)	90(22.28)	
MUOW	632(17.84)	571(18.94)	61(15.12)	
MUO	1077(30.41)	913(26.74)	164(38.54)	
Race				0.434
White	1123(31.71)	973(55.80)	150(59.35)	
Black	850(24)	751(13.50)	99(13.10)	
Other Race	1569(44.3)	1389(30.69)	180(27.55)	
Education level				0.905
Less than high school	695(19.62)	612(18.93)	83(18.85)	< 0.001
High school	538(15.19)	476(11.04)	62(10.31)	< 0.001
More than high school	2309(65.19)	2025(70.03)	284(70.84)	< 0.001
Drinking status				0.199
Yes	2131(60.16)	1881(68.27)	250(64.73)	
No	1411(39.84)	1232(31.73)	179(35.27)	
Smoking status				0.146
Current smoker	612(17.28)	524(17.86)	88(18.89)	
Former smoker	400(11.29)	339(12.84)	61(16.59)	
Never smoker	2530(71.43)	2250(69.30)	280(64.52)	
Physical activity				0.161
High intensity physical	1526(43.08)	1362(47.59)	164(41.76)	
Low intensity physical activity	1063(30.01)	925(29.66)	138(32.48)	
No physical activity	953(26.91)	826(22.75)	127(25.75)	
Irregular Periods	250(7.058)	222(7.505)	28(9.499)	0.359
Married or Living with partner	2032(57.37)	1719(57.95)	313(77.21)	< 0.001
Ever pregnant	2580(72.84)	2224(66.10)	356(84.26)	< 0.001

Abbreviations:

BMI, body mass index; WC, waist circumstance; PIR, poverty income ratio; MHN, metabolic healthy normal weight; MHOW, metabolic healthy overweight; MHO, metabolic healthy obesity; MUN, metabolic unhealthy normal weight; MUOW, metabolic unhealthy overweight; MUO, metabolic unhealthy obesity

### Statistical analysis

Analyses were performed on weighted samples, which permits correction for the over- or under-representation of survey respondents and generalization to the US population. χ2 test (categorical variables), Wilcoxon Rank Sum Test (continuous variables with nonnormal distribution) and T-test (continuous variables with normal distribution) were conducted to test for differences between fertility and infertility groups. Continuous variables were described using the mean and standard error, while categorical variables were described as frequency and percentages. Multiple imputation was conducted to reduce the sample size reduction caused by missing covariates. The principal analysis included five steps. First, we used three logistic regression models: (1) an unadjusted model, (2) an adjusted model containing age, and (3) an adjusted model including all covariables in Table 1(except for menstrual irregularities) to assess the association of BMI and WC with infertility separately. Trend tests were performed by treating the BMI/WC categories as continuous variables. Each group was assigned a median BMI/WC value to generate these categories. Second, the three logistic regression models mentioned previously were employed to estimate the odds ratios (ORs) and 95% confidence intervals (CIs) of infertility among participants across different phenotypes. Third, to investigate the dose-response relationship between BMI/WC and infertility in metabolically healthy and unhealthy participants, we utilized weighted restricted cubic spline (RCS) analysis. In the spline models, adjustments were made for all covariates. Fourth, we used the receiver operator characteristic (ROC) curve to test the accuracy of BMI and WC in predicting infertility among metabolically healthy and unhealthy participants, respectively. Fifth, we conducted two sensitivity analyses: restricting to women aged 27(the average age at first birth in the US)-35 years and excluding those with missing covariate data. We assumed younger women may not have attempted pregnancy and older women may experience weight change during infertility period. We performed all analyses using R (version 4.12). The nhanesR package (v. 0.9.2.3) was utilized to access NHANESR data, whereas the survey package (version 4.1.1) was used to analyze complex survey samples. Two-sided *P* < 0.05 was statistically significant.

## Results

### Baseline characteristics

The inclusion and exclusion criteria are shown in Fig. 1. 3542 individuals were included in our study, representing 36,949,379 women aged 20–45 in the US. 429 infertile women represented a population of 4,571,734 women. The estimated self-reported infertility rate in the US aged 20–45 was 12.11%. Table 1 shows the baseline characteristics of participants included in this study. Infertile participants had a higher BMI and WC (32.12 vs. 29.14, *P* < 0.001; 103.17 vs. 95.01, *P* < 0.001, respectively). And the proportions of individuals who were normal weight and overweight according to the BMI criteria were significantly lower among the infertility participants in both metabolically healthy and unhealthy groups (MHN: 4.93 vs. 6.48; MUN: 22.28 vs. 30.25; MHOW: 3.73 vs. 6.72; MUOW: 15.12 vs. 18.94; *P* < 0.001, respectively) while the proportions of those who were obese were significantly higher among the infertility participants (MHO: 15.39 vs. 10.87; MUO: 38.54 vs. 26.74; *P* < 0.001, respectively). In addition, participants with infertility were older (35.13 vs. 31.66, *P* < 0.001), richer (2.93 vs. 2.66, *P* < 0.05), more likely to married or living with a partner (77.21% vs. 57.95%, *P* < 0.001) and tended to have been pregnant (84.26% vs. 66.10%, *P* < 0.001). The infertile and fertile groups did not differ significantly in terms of race, smoking status, drinking status, education level, menstrual irregularities and physical activity.

### Independent association of BMI and WC with infertility

Three binary logistic regression models were constructed to investigate the potential effect of BMI and WC on infertility. Table 2 demonstrates that BMI and WC were positively correlated with infertility in models 1, 2, and 3, regardless of the controlled covariables. In the crude model (model 1), a one-unit increase in BMI or WC was associated with an increased risk of infertility (OR (95% CI): 1.04 (1.02, 1.06), *P* < 0.001; OR (95% CI): 1.02 (1.01, 1.03), *P* < 0.001; respectively). Each unit increase in BMI or WC remained associated with an increased risk of infertility in models 2 (OR (95% CI): 1.04(1.02, 1.06); *P* < 0.001; OR (95% CI): 1.02 (1.01, 1.03); *P* < 0.001; respectively) and 3(OR (95% CI): 1.04(1.02, 1.06); *P* = 0.001; OR (95% CI): 1.02 (1.01, 1.03); *P* < 0.001; respectively). We transformed BMI and WC into categorical variables (BMI: three groups: “Normal,“ “Overweight,“ and “Obese”; WC: three groups: “Normal,“ “Central overweight,“ and “Central obesity”). After adjusting for all covariables, obesity according to the BMI criteria was associated with an increased risk of infertility (OR (95% CI): 1.83 (1.20, 2.77), *P* = 0.006), and central obesity according to the WC criteria was associated with an increased risk of infertility also (OR (95% CI): 2.18(1.40,3.39), *P* < 0.001). Significant trend associations were observed for BMI/WC and infertility (all *P* for trend < 0.05).

**Table 2:** Risk of infertility for BMI and WC

	Model 1		Model 2		Model 3	
	OR (95% CI)	*P* value	OR (95% CI)	*P* value	OR (95% CI)	*P* value
**BMI(Kg/m** ^**2**^ **)**						
Per 1 unit	1.04 (1.02, 1.06)	< 0.001	1.04 (1.02, 1.06)	< 0.001	1.04 (1.02, 1.06)	0.001
**Groups**						
Normal	1.00(Reference)	-	1.00(Reference)	-	1.00(Reference)	-
Overweight	0.99 (0.66, 1.48)	0.967	0.93(0.62,1.38)	0.704	0.92 (0.60, 1.41)	0.694
Obesity	1.94 (1.35, 2.77)	< 0.001	1.75(1.20,2.54)	0.004	1.83 (1.20, 2.77)	0.006
*P* for trend		0.001		0.004		0.005
**WC(cm)**						
Per 1 unit	1.02 (1.01, 1.03)	< 0.001	1.02 (1.01, 1.03)	< 0.001	1.02 (1.01, 1.03)	< 0.001
**Groups**						
Normal (< 80 cm)	1.00(Reference)	-	1.00(Reference)	-	1.00(Reference)	-
Central overweight, (< 88 cm)	1.73(1.07,2.80)	0.03	1.52(0.94,2.45)	0.08	1.46(0.92,2.34)	0.11
Central obesity ( > = 88 cm)	2.71(1.84,4.01)	< 0.001	2.24(1.49,3.38)	< 0.001	2.18(1.40,3.39)	< 0.001
*P* for trend		< 0.001		< 0.001		< 0.001

Abbreviations: BMI, body mass index; WC, waist circumstance; OR, odds ratio; CI, confidence interval.

Note: Model 1: Adjusted for nothing.

Model 2: Adjusted for baseline age.

Model 3: Adjusted for model 2 plus race, marital status, poverty income ratio, drinking status, smoking status, education level, pregnant history, physical activity

### Associations between metabolic health-obesity phenotypes with infertility

The risks for infertility cross-classified by metabolic health and obesity (BMI and WC categories) are presented in Table 3. After adjusting for all covariables, individuals with MHO had a relatively higher risk of infertility than those with MHN (BMI criteria: OR (95% CI): 1.75(0.88, 3.50), *P* = 0.109; WC criteria: OR (95% CI): 2.01(1.03, 3.95), *P* = 0.042). Additionally, the risk of infertility in MUO participants was significantly higher than that in MUN individuals (BMI criteria: OR (95% CI): 1.85(1.15, 2.97), *P* = 0.012; WC criteria: OR (95% CI): 2.24(1.36, 3.70), *P* = 0.002). Each unit increase in BMI or WC was associated with an increased risk of infertility among both metabolic healthy and unhealthy participants (OR (95% CI): 1.06(1.02, 1.09); *P =* 0.004; OR (95% CI): 1.04 (1.01, 1.06); *P* = 0.002; OR (95% CI): 1.03(1.01, 1.04); *P =* 0.007; OR (95% CI): 1.02 (1.01, 1.03); *P* < 0.001; respectively). Significant trend associations were observed for BMI/WC and infertility in both metabolically healthy and unhealthy participants (all *P* for trend < 0.05) except for BMI in the metabolically healthy group (*P* = 0.053).

**Table 3:** Risk of infertility for BMI and WC among metabolic healthy and unhealthy group

Metabolic health obesity phenotypes	n/N	Model 1		Model 2		Model 3	
		OR (95% CI)	*P* value	OR (95% CI)	*P* value	OR (95% CI)	*P* value
**Body Mass Index (kg/m** ^**2**^ **) categories**							
Per 1 unit		1.05(1.02,1.09)	< 0.001	1.05(1.02,1.08)	0.004	1.06(1.02,1.09)	0.004
MHN	25/220	1.00(Reference)	-	1.00(Reference)	-	1.00(Reference)	-
MHOW	19/252	0.73(0.31,1.73)	0.469	0.61(0.25,1.47)	0.263	0.67(0.28,1.64)	0.373
MHO	70/433	1.86(0.97,3.58)	0.063	1.65(0.83,3.28)	0.152	1.75(0.88,3.50)	0.109
*P* for trend			0.034		0.067		0.053
Per 1 unit		1.04(1.02,1.06)	< 0.001	1.03(1.01,1.05)	0.002	1.04(1.01,1.06)	0.002
MUN	90/928	1.00(Reference)	-	1.00(Reference)	-	1.00(Reference)	
MUOW	61/632	1.08(0.73,1.61)	0.684	1.04(0.70,1.54)	0.837	1.01(0.68,1.51)	0.950
MUO	164/1077	1.96(1.32,2.90)	0.001	1.74(1.16,2.63)	0.009	1.85(1.15,2.97)	0.012
*P* for trend			0.002		0.01		0.012
**WC (cm) categories**							
Per 1 unit		1.03(1.01,1.04)	0.001	1.03(1.01,1.04)	0.001	1.03(1.01,1.04)	0.007
MHLW	11/131	1.00(Reference)	-	1.00(Reference)	-	1.00(Reference)	-
MHMW	15/143	1.74(0.75,4.03)	0.190	1.43(0.65,3.19)	0.368	1.40(0.63,3.13)	0.398
MHO	88/631	2.55(1.35,4.84)	0.005	1.98(1.00,3.92)	0.049	2.01(1.03,3.95)	0.042
*P* for trend			0.006		0.063		0.05
Per 1 unit		1.02(1.01,1.03)	< 0.001	1.02(1.01,1.03)	< 0.001	1.02(1.01,1.03)	< 0.001
MULW	42/549	1.00(Reference)	-	1.00(Reference)	-	1.00(Reference)	
MUMW	48/494	1.73(0.95,3.15)	0.075	1.54(0.85,2.80)	0.153	1.50(0.84,2.68)	0.167
MUO	225/1594	2.78(1.79,4.32)	< 0.001	2.31(1.45,3.67)	< 0.001	2.24(1.36,3.70)	0.002
*P* for trend			< 0.001		< 0.001		0.002

Abbreviations: BMI, body mass index; WC, waist circumstance; OR, odds ratio; CI, confidence interval; MHN, metabolic healthy normal weight; MHOW, metabolic healthy overweight; MHO, metabolic healthy obesity; MUN, metabolic unhealthy normal weight; MUOW, metabolic unhealthy overweight; MUO, metabolic unhealthy obesity.

Note: Model 1: Adjusted for nothing.

Model 2: Adjusted for baseline age.

Model 3: Adjusted for model 2 plus race, marital status, poverty income ratio, drinking status, smoking status, education level, pregnant history, physical activity.

### Restricted cubic spline

**Fig. 2:**
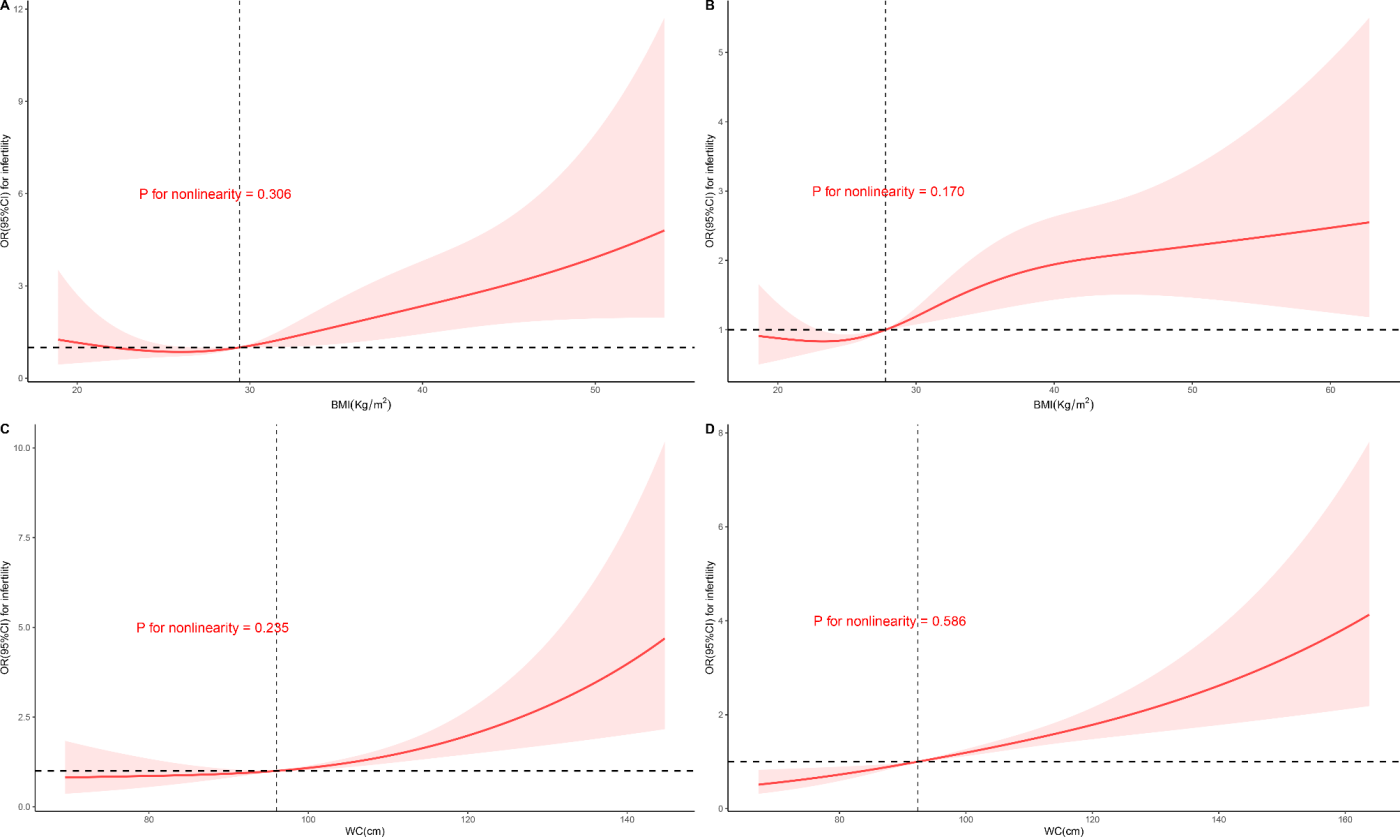
Restricted cubic spline model of the association of BMI/WC and infertility among the metabolic healthy and unhealthy group. **Note**: A: Metabolic healthy group using BMI criteria. B: Metabolic unhealthy group using BMI criteria. C: Metabolic healthy group using WC criteria. D: Metabolic unhealthy group using WC criteria. The red curve and the pink area represent the odds ratio and 95% confidence interval, respectively. The black horizontal dashed line indicates the odds ratio = 1. The black vertical dashed line indicates the reference, which equals to medium BMI/WC of each group

We utilized RCS to simulate and model the relation of BMI and WC with infertility among metabolically healthy and unhealthy participants (Fig. 2). The dose-response relationship between BMI/WC and infertility was approximately linear (all *P* for non-linearity > 0.05) throughout the range of their levels in whether metabolically healthy or metabolically unhealthy participants, indicating a positive association between BMI/WC and infertility.

### Receiver operator characteristic curve

The area under the ROC curve (AUC) of the BMI among metabolically healthy participants for predicting the occurrence of infertility was 0.576 (95%CI: (0.517,0.634)), whereas the AUC of BMI among metabolically unhealthy participants was 0.581 (95%CI (0.547,0.616)). In addition, the AUC of WC among metabolically healthy participants was 0.583 (95%CI: (0.525,0.641)), while the AUC of WC among metabolically unhealthy participants was 0.599 (95%CI: (0.566,0.633))(Supplemental Fig. 2). No significant differences was observed between the predicated ability of WC/BMI in metabolically healthy participants or metabolically unhealthy participants (BMI criteria: P_MH vs. MUH_ = 0.865; WC criteria: P_MH vs. MUH_ = 0.640).

### Sensitivity analyses

Sensitivity analyses were presented in Supplemental Table 1. The associations of the MHO and MUO phenotype with infertility were nearly unchanged when restricting participants aged 27–35 years old and excluding covariates with missing values.

## Discussion

In this nationally representative cross-sectional study, we examined the associations between obesity and infertility among metabolically healthy and unhealthy women aged 20 to 45. According to the weighted analysis, the prevalence of infertility among 20-45-year-old women was 12.11%, matching the predicted national prevalence of 12–18% [25].

We present numerous significant findings in this study. First, a high BMI and WC were related to an increased risk of infertility after controlling for conventional risk factors. Second, after dividing the population into metabolic healthy and unhealthy, the increased risk of infertility remained in both MHO and MUO phenotypes. Third, BMI and WC were positively associated with the risk of infertility in a linear dose–response manner among both metabolic healthy and unhealthy women, which further confirmed that MHO is not a benign condition and is associated with an increased risk of infertility. Lastly, the ROC curve showed that BMI and WC predicted infertility with no difference between metabolic healthy and unhealthy women.

The association between obesity and infertility has been well-established in many studies [26, 27]. However, obesity also promotes insulin resistance and related metabolic abnormalities that promote infertility [28–30]. Thus, it is unclear if obesity alone causes infertility, or together with metabolic disorders. Stratifying women by both obesity and metabolic health may clarify obesity’s role. Our discovery that the risk of infertility remained elevated in the MHO population implies that obesity is a separate risk factor for infertility.

The connection between obesity and infertility has biological and social-psychological bases. Biologically, the effects of excess body fat on sex hormone secretion and metabolism are profound [11]. Adiposity may increase luteinizing hormone levels, raise peripheral aromatization of androgens to estrogens, and decrease the synthesis of sex hormone-binding globulin (SHBG) in the liver, which may interface the hypothalamic-pituitary-ovary axis, causing follicular atresia and anovulatory cycles [31–33]. The distribution of body fat has a significant impact on hormone concentration as well. Regardless of BMI value, a negative connection exists between central obesity and testosterone or SHBG concentrations [34]. The mechanism by which these factors interact with folliculogenesis remains unknown; nonetheless, it is evident that obesity has a direct and adverse effect on fertility. In addition, obesity appears to affect the oocyte and the preimplantation embryo, with disrupted meiotic spindle formation and mitochondrial dynamics. What’ s more, excess free fatty acids may have a toxic effect in reproductive tissues, leading to cellular damage and a chronic low-grade inflammatory state [35]. All of these factors contributed to infertility. Social psychologically, obesity can also alter the sexual desire and frequency of sexual life, negatively impacting fertility [36].

Our investigation also confirms that BMI or WC predicted infertility with no difference among metabolic healthy and unhealthy women, which suggests that obesity plays a similar role in both groups, indicating that MHO is also a risk factor for infertility once again. It advises that obesity should be avoided regardless of metabolic disorders.

Also, as expected, obesity associated with higher infertility risk in metabolically unhealthy women. This reaffirms that obesity should be avoided, whether metabolically healthy or unhealthy.

To our knowledge, this study first examined the association between MHO and female infertility using participants from a large, nationally representative population. We accounted for various covariates that might modulate infertility and obesity. Our resreach included women aged 20–45 years, though we lacked information about the causes of infertility. Therefore, it is possible to apply the findings of this study to a comparable population of 20-45-year-old American women. Finally, we conducted two sensitivity analyses to confirm the robustness of the conclusions.

The conclusions of this study must be interpreted in light of several limitations. First, since this is a cross-sectional study, causal judgments regarding fat and infertility cannot be made. Second, we lack information regarding the duration of infertility, which could modulate BMI during this period. Third, infertility was self-reported; the underlying causes are unknown. We cannot ascribe obesity to infertility in all cases. Fourth, our study could have missed women who might be infertile but have not tried to conceive yet.

## Conclusions

MHO was associated with high infertility risk among reproductive-aged women in the US. Obesity relates to greater infertility risk, regardless of metabolic health. Our findings support lifestyle changes to achieve and maintain a healthy weight for all, even those metabolically healthy. To confirm our results, further prospective epidemiological studies are needed.

### Electronic supplementary material

Below is the link to the electronic supplementary material.

Supplementary Material 1

Supplementary Material 2

Supplementary Material 3

Supplementary Material 4

## Data Availability

Publicly available datasets were analyzed in this study. This data can be found here: https://wwwn.cdc.gov/nchs/nhanes/Default.aspx (assessed on 15 July 2022).
