# Supplementary material for: Association between metabolic healthy obesity and female infertility: the national health and nutrition examination survey, 2013–2020
Source: BMC Public Health. 2023 Aug 10;23:1524. doi: 10.1186/s12889-023-16397-x (PMC10416469; doi:10.1186/s12889-023-16397-x)
Supplement: Supplementary file 1 — Supplementary Material 1 [file 12889_2023_16397_MOESM1_ESM.docx]

**Supplemental Figure Legends**

**Supplemental figure 1** Directed acyclic graph

Note: A directed acyclic graph represents associations between covariates and primary exposure and outcome. Pink circles represent confounders, blue circles represent ancestors of the outcome (i.e., causal determinants of the outcome), and green circles represent the outcome. Yellow circles represent mediators. Diabetes mellitus and irregular menstruation were considered to be mediators. The final minimally sufficient adjustment set comprised age, race, marital status, education level, RIP(ratio of family income to poverty), drinking status, smoking status, pregnancy history, and PA(physical activity).

**Supplemental figure 2** The ROC curve of BMI and WC among the metabolic healthy and unhealthy group
