# Supplementary figures and images for: Association between metabolic healthy obesity and female infertility: the national health and nutrition examination survey, 2013–2020
Source: BMC Public Health. 2023 Aug 10;23:1524. doi: 10.1186/s12889-023-16397-x (PMC10416469; doi:10.1186/s12889-023-16397-x)

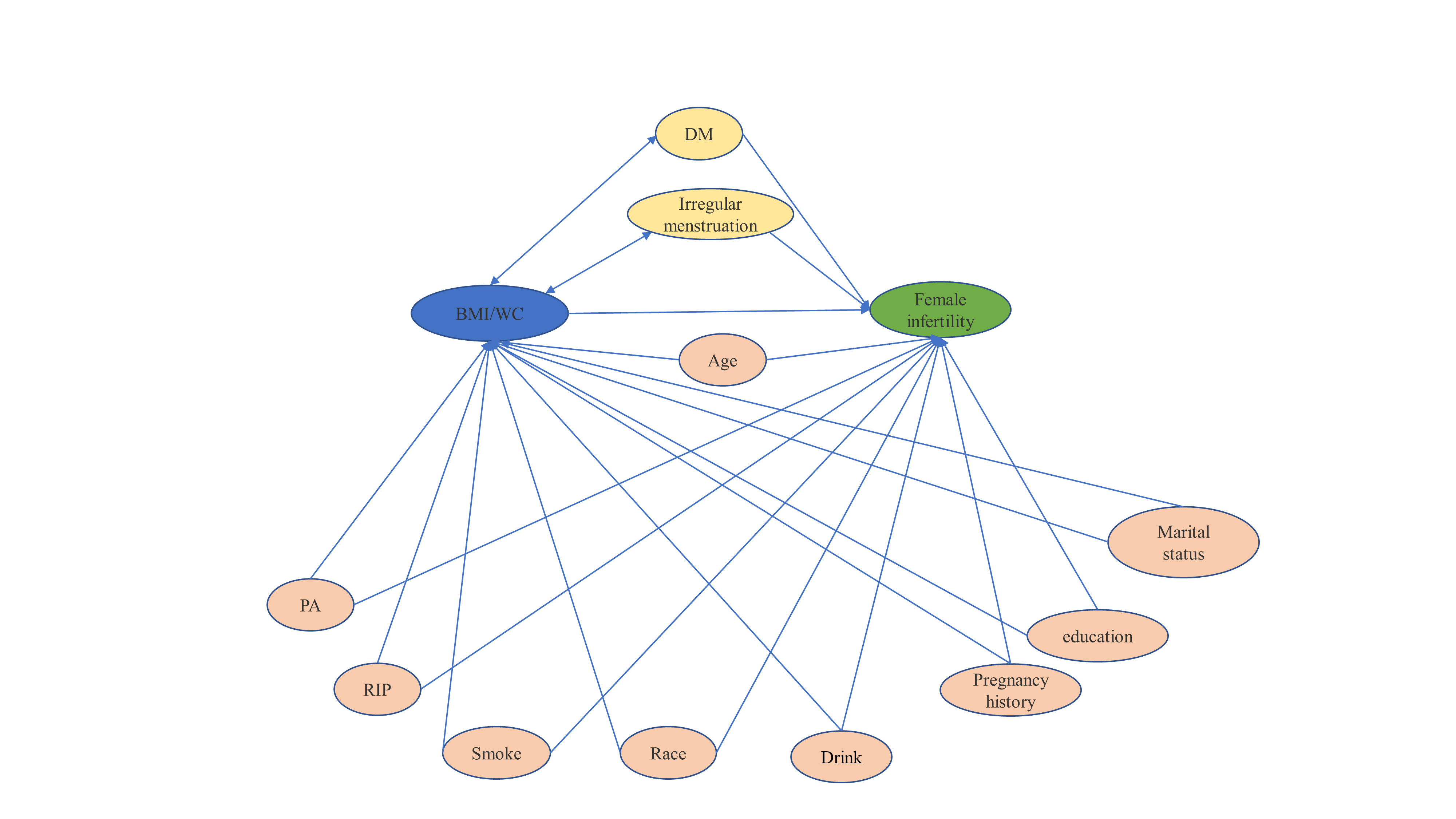

Supplement: Supplementary file 2 — Supplementary Material 2 [file 12889_2023_16397_MOESM2_ESM.png]

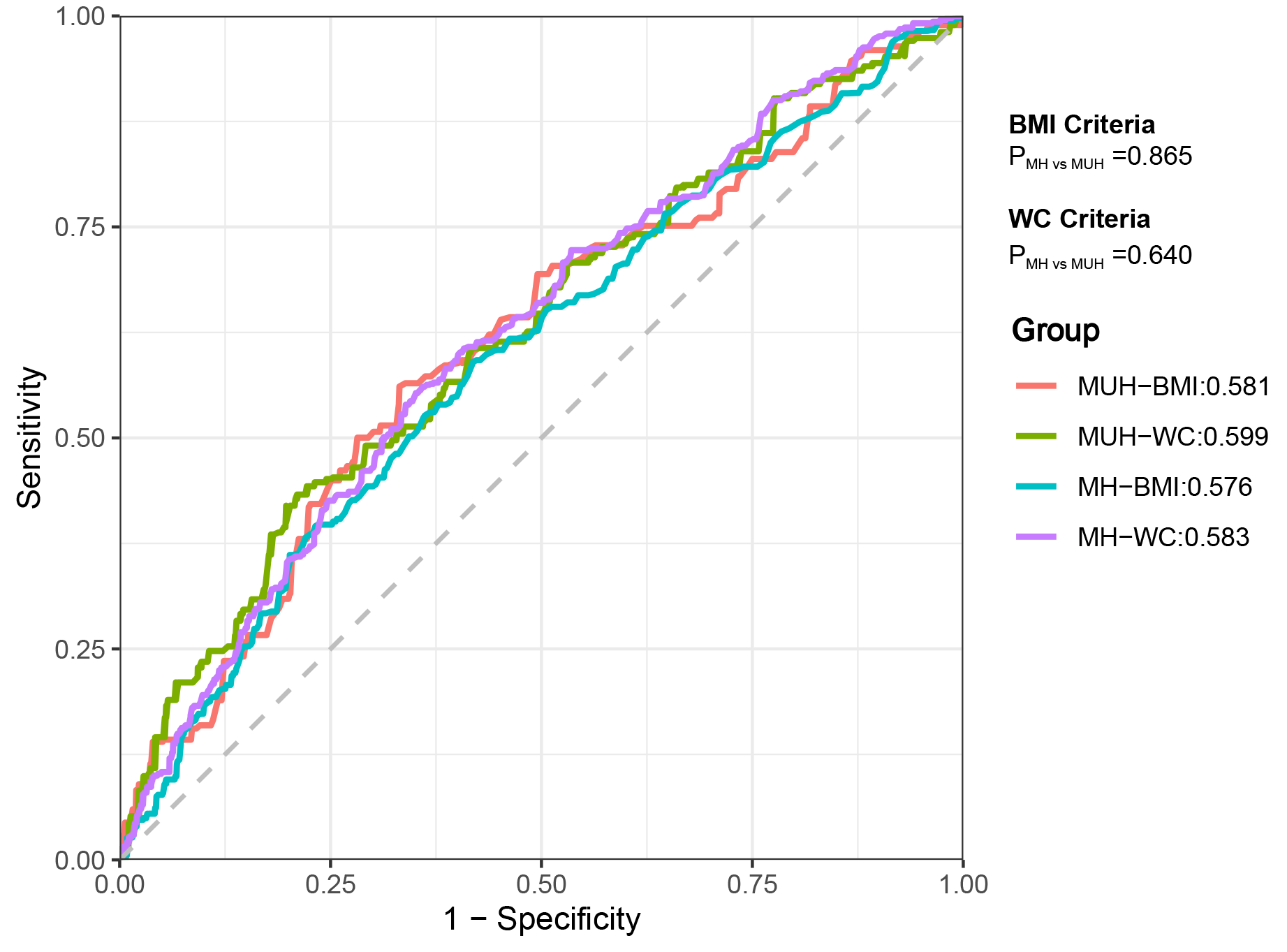

Supplement: Supplementary file 3 — Supplementary Material 3 [file 12889_2023_16397_MOESM3_ESM.png]
