# Supplementary material for: Association between metabolic healthy obesity and female infertility: the national health and nutrition examination survey, 2013–2020
Source: BMC Public Health. 2023 Aug 10;23:1524. doi: 10.1186/s12889-023-16397-x (PMC10416469; doi:10.1186/s12889-023-16397-x)
Supplement: Supplementary file 4 — Supplementary Material 4 [file 12889_2023_16397_MOESM4_ESM.docx]

**Supplemental table 1. Sensitivity analyses of the association between BMI/WC and infertility among metabolic healthy and unhealthy group**

| **Metabolic health**  **obesity phenotypes** | **Restricting participants Between 27-35 years old (N=1243)** |  | **Excluding covariates**  **with missing values (N=2946)** |  |
| --- | --- | --- | --- | --- |
|  | **OR (95% CI)** | ***P* value** | **OR (95% CI)** | ***P* value** |
| **Body Mass Index (kg/m^2^) categories** | | | | |
| Per 1 unit | 1.04(1.00,1.08) | 0.059 | 1.06(1.02,1.10) | 0.004 |
| MHN | 1.00(Reference) | - | 1.00(Reference) | - |
| MHOW | 0.97(0.27,3.48) | 0.968 | 0.75(0.27,2.09) | 0.575 |
| MHO | 2.54(0.94,6.80) | 0.064 | 1.95(0.88,4.30) | 0.097 |
| *P* for trend |  | 0.035 |  | 0.047 |
| Per 1 unit | 1.04(1.01,1.07) | 0.016 | 1.04(1.01,1.06) | 0.003 |
| MUN | 1.00(Reference) | - | 1.00(Reference) |  |
| MUOW | 0.76(0.37,1.54) | 0.433 | 0.92(0.58,1.45) | 0.708 |
| MUO | 1.77(0.94,3.36) | 0.078 | 1.93(1.19,3.13) | **0.009** |
| *P* for trend |  | 0.073 |  | **0.009** |
| **WC (cm) categories** | | | | |
| Per 1 unit | 1.02(1.00,1.04) | 0.044 | 1.03(1.01,1.05) | 0.009 |
| MHLW | 1.00(Reference) | - | 1.00(Reference) | - |
| MHMW | 1.16(0.24,5.50) | 0.847 | 2.43(0.89,6.69) | 0.083 |
| MHO | 1.87(0.63,5.61) | 0.252 | 3.35(1.29,8.69) | **0.014** |
| *P* for trend |  | 0.181 |  | **0.023** |
| Per 1 unit | 1.02(1.01,1.04) | <0.001 | 1.02(1.01,1.03) | <0.001 |
| MULW | 1.00(Reference) | - | 1.00(Reference) |  |
| MUMW | 1.19(0.40,3.50) | 0.747 | 1.65(0.88,3.09) | 0.112 |
| MUO | 2.71(1.22,5.99) | 0.015 | 2.32(1.33,4.05) | 0.004 |
| *P* for trend |  | **0.005** |  | 0.005 |

**Abbreviations:** BMI, body mass index; WC, waist circumstance; OR, odds ratio; CI, confidence interval; MHN, metabolic healthy normal weight; MHOW, metabolic healthy overweight; MHO, metabolic healthy obesity; MUN, metabolic unhealthy normal weight; MUOW, metabolic unhealthy overweight; MUO, metabolic unhealthy obesity.

**Note:** Adjusted for age, race, marital status, poverty income ratio, drinking status, smoking status, education level, pregnant history, physical activity.
